# Upright supine imaging can detect clinically relevant occult instability in degenerative lumbar spondylolisthesis - A two-year PROM based comparison with flexion extension radiographs

**DOI:** 10.1016/j.bas.2026.106064

**Published:** 2026-04-23

**Authors:** Tom Folkerts, Julia Wimmer, Lukas Schönnagel, Anna-Maria Mielke, Bruno Verna, Pedro Rocha Torres, Paul Köhli, Jiaqi Zhu, Jennifer Shue, Rolando Duculan, Andrew A. Sama, Federico P. Girardi, Frank P. Cammisa, Carol A. Mancuso, Marco D. Burkhard, Alexander P. Hughes

**Affiliations:** a Center for Musculoskeletal Surgery, Charité - Universitätsmedizin Berlin, Berlin, Germany; b Department of Orthopaedic Surgery, Hospital for Special Surgery, Weill Cornell Medicine, New York City, NY, USA; c Center for Biostatistics, Hospital for Special Surgery, New York City, NY, USA; d Department of Medicine, Hospital for Special Surgery, Weill Cornell Medicine, New York City, NY, USA

**Keywords:** Degenerative spondylolisthesis, Decompression surgery, Lumbar spine, Segmental instability, Flexion extension radiographs, Upright supine imaging

## Abstract

**Introduction:**

Flexion-extension radiographs (FER) are widely used to assess segmental instability in degenerative lumbar spondylolisthesis (DLS) and guide surgical decision-making, as dynamic instability may be associated with suboptimal outcomes after decompression only. However, FER rely on patient effort, and pain or limited mobility can reduce reproducibility and underestimate instability. Upright-supine imaging (USI), comparing upright radiographs with supine MRI, may better capture slip dynamics, but its clinical value remains undefined.

**Research question:**

To evaluate whether USI-derived segmental instability provides additional prognostic information beyond FER for predicting postoperative improvement in disability and low back pain (LBP) following decompression-only surgery for DLS.

**Material and methods:**

This retrospective analysis of prospectively collected data included 92 DLS patients undergoing decompression-only surgery. Slip dynamics were quantified as between-position difference in relative translation (ΔRT) on FER and USI. Multivariable regression assessed associations between ΔRT and two-year outcomes (Oswestry Disability Index [ODI], LBP numeric rating scale). ROC analyses determined optimal ΔRT_USI cutoffs.

**Results:**

ΔRT_USI independently predicted postoperative improvement in ODI (p < 0.001) and LBP (p < 0.001), whereas ΔRT_FER was not associated with outcomes. ROC analysis identified a ΔRT_USI threshold of 7.9% as optimal (ODI: AUC = 0.87, sensitivity = 0.86, specificity = 0.81; LBP: AUC = 0.62, sensitivity = 0.9, specificity = 0.48). USI identified instability in 22.8% of patients, uncovering occult instability that FER missed. USI-unstable patients showed significantly less postoperative improvement (p < 0.001).

**Discussion and conclusion:**

USI detects clinically relevant occult instability missed by FER and independently predicts outcomes after decompression-only surgery. A ΔRT_USI threshold of approximately 8% serves as a quantitative marker supporting USI for preoperative assessment in DLS.

## Highlights

• ΔRT_USI predicted 2-year ODI and LBP improvement after decompression-only.
• ΔRT_FER was not associated with postoperative outcomes.
• USI revealed instability missed by FER in 22.8% of patients.
• A 8% ΔRT_USI cutoff identified patients at risk for inferior improvement.

## List of abbreviations

AUC: Area Under the Curve
BMI: Body Mass Index
DLS: Degenerative Lumbar Spondylolisthesis
FER: Flexion-Extension Radiographs
ICC: Intraclass Correlation Coefficient
IQR: Interquartile Range
LBP: Low Back Pain
ΔLBP_abs: Absolute pre-to postoperative Difference in Low Back Pain
ΔLBP_rel: Relative pre-to postoperative Difference in Low Back Pain
MCID: Minimal Clinically Important Difference
MRI: Magnetic Resonance Imaging
NRS: Numeric Rating Scale
ODI: Oswestry Disability Index
ΔODI_abs: Absolute pre-to postoperative Difference in Oswestry Disability Index
ΔODI_rel: Relative pre-to postoperative Difference in Oswestry Disability Index
PROM: Patient-Reported Outcome Measure
ROC: Receiver Operating Characteristic
RT: Relative Translation
RT_FER: Relative translation on Flexion-Extension Radiographs
RT_USI: Relative translation on Upright-Supine Imaging
ΔRT: Absolute Difference in Relative Translation
ΔRT_FER: Absolute Difference in Relative Translation on Flexion-Extension Radiographs
ΔRT_USI: Absolute Difference in Relative Translation on Upright-Supine Imaging
S: Supine
U: Upright
USI: Upright-Supine Imaging
VIF: Variance Inflation Factor

## 1 Introduction

Degenerative lumbar spondylolisthesis (DLS) is characterized by an acquired vertebral subluxation of a lumbar vertebra relative to the subjacent segment, driven by progressive degenerative changes (Saremi et al., 2024; Matz et al., 2016). This degenerative process alters spinal biomechanics by redistributing axial load toward the facet joints, thereby promoting segmental instability and inducing secondary structural changes such as facet joint hypertrophy, ligamentum flavum thickening, and progressive narrowing of the spinal canal and foramina (Saremi et al., 2024; Rangwalla et al., 2024; Kalichman and Hunter, 2008; Lee and Patel, 2013). The resulting low back pain (LBP), radicular symptoms, and neurogenic claudication render DLS a leading indication for lumbar spine surgery, when conservative measures fail (Denard et al., 2010; Jacobsen et al., 2007; Spiker et al., 2019; Fujimori et al., 2015; Bond et al., 2020).

Despite its clinical significance, there is no universal consensus on the optimal surgical management of DLS, largely due to the heterogeneity in clinical presentation, radiographic characteristics, and surgeons’ preferences (Matz et al., 2016; Cabrera et al., 2024; Schneider et al., 2021; Hellum et al., 2023; Austevoll et al., 2021; Försth et al., 2016; Ghogawala et al., 2016). While the overarching goal is to alleviate pain and improve function, a broad spectrum of surgical strategies exists that range from decompression alone to various fusion techniques (Cabrera et al., 2024; Schöller et al., 2017). A key factor guiding the surgical decision-making is the degree of dynamic instability. Patients with pronounced instability may benefit more from fusion, given that decompression alone does not address the mechanical component of pain and can further increase slip dynamics. Whereas in the absence of dynamic instability, decompression alone may achieve favorable outcomes (Cabrera et al., 2024; Reijmer et al., 2024; Strube et al., 2019; Morse et al., 2022; Burkhard et al., 2023; Schönnagel et al., 2024).

While flexion-extension radiographs (FER) are considered the diagnostic standard for assessing dynamic segmental instability in DLS, their use is associated with additional healthcare costs and radiation exposure, with a two-view lumbar FE series delivering an estimated effective dose of approximately 2.2 mSv (Cabrera et al., 2024; Liu et al., 2015; Mellor et al., 2014). Most notably, limited reproducibility and restricted applicability in patients with pain or reduced mobility may lead to an underestimation of instability, thereby impairing surgical decision-making and postoperative results (Cabrera et al., 2024; Liu et al., 2015; Kashigar et al., 2021; Schroeder et al., 2015). Given these limitations, upright-supine imaging (USI) that combines upright (U) lateral radiographs with supine (S) magnetic resonance imaging (MRI), has been proposed as a less invasive and clinically accessible alternative for detecting dynamic instability in DLS. However, despite prior studies suggesting that this combination may be equivalent or even superior to FER in detecting lumbar instability, its clinical relevance for predicting postoperative outcomes remains unclear (Liu et al., 2015; Kashigar et al., 2021; Chan et al., 2019; Viswanathan et al., 2018; Folkerts et al., 2025).

Therefore, the present study aimed to evaluate the prognostic value of USI-derived segmental stability in patients undergoing decompression-only surgery for DLS and to determine whether it provides additional information for predicting postoperative improvement in disability and LBP beyond FER-based evaluation. We hypothesized that USI is capable of detecting clinically relevant occult instability that may not be identified on conventional FER. Secondly, we hypothesized that patients with USI-defined instability experience less postoperative improvement in functional disability and LBP after decompression-only.

## 2 Materials and methods

### 2.1 Study design

This study is a single-center retrospective analysis of a prospectively collected consecutive cohort of patients who underwent lumbar decompression-only surgery for symptomatic DLS between January 2016 and December 2018 at a tertiary care academic spine center. This study adhered to the Strengthening the Reporting of Observational Studies in Epidemiology (STROBE) guidelines (Cuschieri, 2019; Vandenbroucke et al., 2007). It was approved by the Institutional Review Board at our institution (IRB# 2015-237) and all patients provided written informed consent.

All operations were performed by board-certified attending spine surgeons, each with at least ten years of experience. Procedures consisted of open posterior decompression via laminectomy through a standard posterior approach. Postoperative care followed standardized institutional protocols and best practice guidelines. Patient-reported outcome measures (PROMs), including the Oswestry Disability Index (ODI) and Numeric Rating Scale (NRS) for LBP, were prospectively collected preoperatively and at two-year follow-up.

### 2.2 Data collection and population

The study cohort included adults (age ≥18 years) with a diagnosis of DLS who underwent decompression-only surgery without fusion. Eligible participants were required to be able to provide informed consent and to understand English sufficiently to complete the PROMs questionnaires. Inclusion required complete preoperative imaging of the lumbar spine, consisting of an U standing lateral radiograph, a S MRI scan, and dynamic weight-bearing FER obtained in the standing position, as well as complete pre- and postoperative PROMs. Patients were excluded if they had a history of prior lumbar surgery, missing or poor-quality preoperative imaging, or incomplete pre- or postoperative PROMs.

### 2.3 Assessment of slip dynamics and instability

**Fig. 1:**
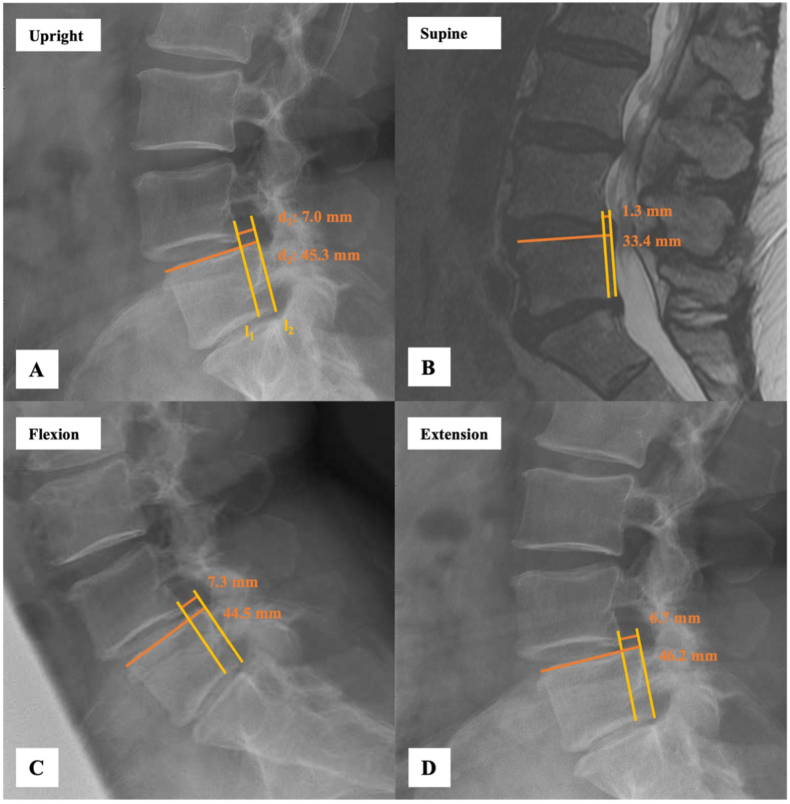
Sagittal translation in a 60-year-old patient with L4–5 degenerative spondylolisthesis, demonstrating position-dependent variation in slip dynamics across upright (A), supine (B), flexion (C), and extension (D) imaging. In (A), sagittal translation (d_1_) is measured between parallel lines l_1_ (posterior margin of L5) and l_2_ (posterior inferior corner of L4), while vertebral body width (d_2_) is measured along the superior endplate of L5.^31^ The difference in relative translation between flexion and extension is 1.9%, whereas the difference between upright and supine amounts to 11.6%.

Slip dynamics was defined as the difference in relative translation (ΔRT) between two imaging positions, either flexion and extension or upright (U) and supine (S), representing the position-dependent translation of the superior vertebra relative to the adjacent inferior vertebra at the spondylolisthesis level (Fig. 1). For MRI measurements, the midsagittal slice at the level of the DLS was selected by first identifying the geometric center of the affected vertebral body and spinal canal on axial images. The relative translation (RT) was then measured on the corresponding sagittal plane, where the spinous processes and vertebral margins were clearly delineated. Assessment of RT was performed by measuring the absolute sagittal translation (d_1_) and the anteroposterior length of the superior endplate of the caudal vertebral body (d_2_) on standing lateral radiographs of the lumbar spine (Fig. 1A). The absolute translation (d_1_) was determined by drawing a reference line (l_1_) along the posterior margin of the inferior vertebral body and a second, parallel line (l_2_) along the posterior margin of the superior vertebral body. The perpendicular distance between these two lines represented d_1_, measured in millimeters. To account for interindividual differences in vertebral body size, the anteroposterior length of the superior endplate of the caudal vertebra (d_2_) was measured in the same image. The RT was then calculated as the ratio of d_1_ to d_2_ according to the following formula (Liu et al., 2015; Folkerts et al., 2025; Köhli et al., 2024).

I. Relative Translation (RT) [%] = (d_1_ [mm]/d_2_ [mm]) × 100%.

According to previous studies based on FER, segmental instability was defined using an established threshold of ≥8 percentage points in relative translation (ΔRT_FER) to distinguish FER-stable from FER-unstable segments (Liu et al., 2015; Boden and Wiesel, 1990). This 8% cutoff, however, has been validated exclusively for FER. No corresponding, clinically validated threshold exists for USI (ΔRT_USI) measurements. Therefore, we performed a receiver operating characteristic (ROC) analysis to identify a clinically meaningful ΔRT_USI cutoff to predict the minimal clinically important difference (MCID) in functional disability and LBP. This data-driven threshold was subsequently used to classify segments as USI-stable or USI-unstable.

### 2.4 Patient-reported outcome measures

The collected PROMs included pre- and two-years postoperative measures of functional disability, assessed using the ODI, and LBP intensity rated on the NRS.

The ODI is a validated 10-item questionnaire assessing disability related to spinal disorders, covering domains such as pain, mobility, and daily activities. Each item is scored from 0 to 5, with total scores converted to a percentage from 0% (no disability) to 100% (maximum disability). Both the absolute (ΔODI_abs = preoperative ODI – postoperative ODI) and relative (ΔODI_rel = ΔODI_abs/preoperative ODI × 100%) changes were calculated. An improvement of ≥10 absolute ODI points was defined as the minimal clinically important difference (MCID) (Fairbank et al., 1980; Hung et al., 2018).

LBP intensity was evaluated using the NRS, a validated 11-point scale ranging from 0 (“no pain”) to 10 (“worst imaginable pain”). Preoperative and two-year postoperative NRS scores were recorded, and both the absolute (ΔLBP_abs = preoperative NRS – postoperative NRS) and relative (ΔLBP_rel = ΔNRS_abs/preoperative NRS × 100%) changes were calculated. A reduction of ≥2 absolute NRS points was defined as the MCID (Farrar et al., 2001; Chiarotto et al., 2019).

### 2.5 Statistical analysis

Prior to analysis, continuous variables were assessed for normal distribution using the Shapiro-Wilk test. Variables are reported as medians (interquartile range [IQR]), whereas categorical variables are presented as absolute numbers (=N). Between-group differences were evaluated with a Mann–Whitney *U* test for nonparametric data, whereas categorical variables were analyzed using the Chi-square test. Multivariable linear regression analyses were conducted to assess the association between ΔRT (USI and FER) and postoperative improvement in functional disability (ΔODI_rel) and LBP (ΔLBP_rel). Models were adjusted for age, sex, body mass index (BMI) and number of levels decompressed, as these factors have been linked to postoperative improvement and dynamic instability (Garcia et al., 2025; Elsamadicy et al., 2017; Zammar et al., 2024; Gonzalez et al., 2022; Lee et al., 2020; Nakajima et al., 2023). Multicollinearity was evaluated using the variance inflation factor (VIF), with values < 5 considered acceptable. The coefficient of determination (R^2^) was reported for each model to indicate the proportion of explained variance in the dependent variable. Radiographic images were independently evaluated by two spine surgeons, each blinded to the other's measurements. Interrater reliability for radiographic assessment of RT was evaluated using the intraclass correlation coefficient (ICC). To identify a clinically relevant threshold of USI translation predictive of meaningful postoperative improvement, ROC curve analysis was performed. The area under the curve (AUC) and the optimal cutoff point for predicting achievement of the MCID were determined using the Youden Index, defined as the point maximizing the sum of sensitivity and specificity (Ruopp et al., 2008). All analyses were performed using RStudio (Posit Software, Boston, MA). Statistical significance was set at p < 0.05.

## 3 Results

### 3.1 Patient characteristics

**Fig. 2:**
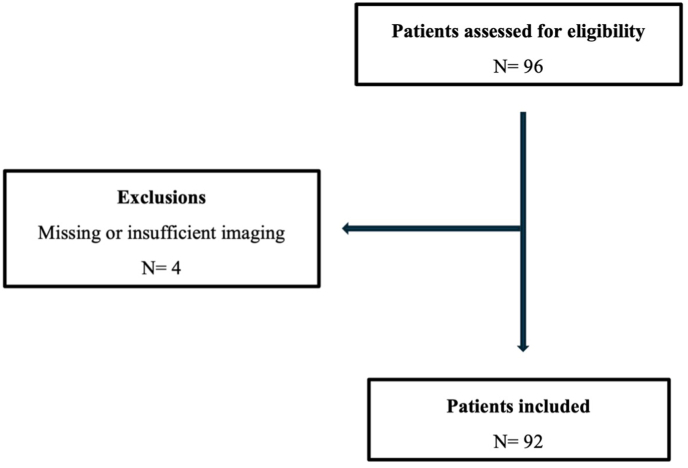
Flow chart of patient inclusion.

A total of 96 patients underwent decompression-only surgery for DLS. Four patients were excluded due to missing or insufficient imaging, leaving 92 patients for the final analysis (Fig. 2). Forty patients (43.5%) were female, the median age was 69.7 years (IQR 64.9-74.2), and the median BMI was 28.8 kg/m^2^ (IQR 26.4-33.5). All cases of DLS were Meyerding grade I, and L4-5 was the most frequently affected level (92.4%).

### 3.2 Association of USI- and FER-derived translation with ODI and LBP improvement

**Fig. 3:**
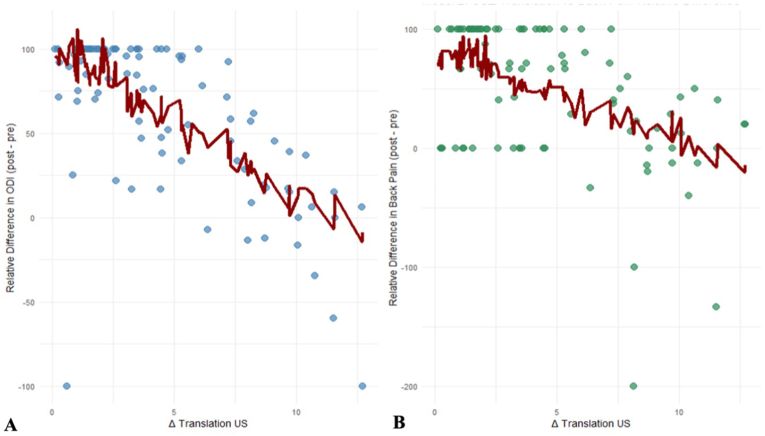
Relationship between upright–supine imaging (USI) translation and relative postoperative improvement in (A) functional disability (ODI) and (B) low back pain (LBP). Each dot represents an individual patient. The red line shows the predicted postoperative improvement based on the fitted regression model for the observed USI translation values.

**Fig. 4:**
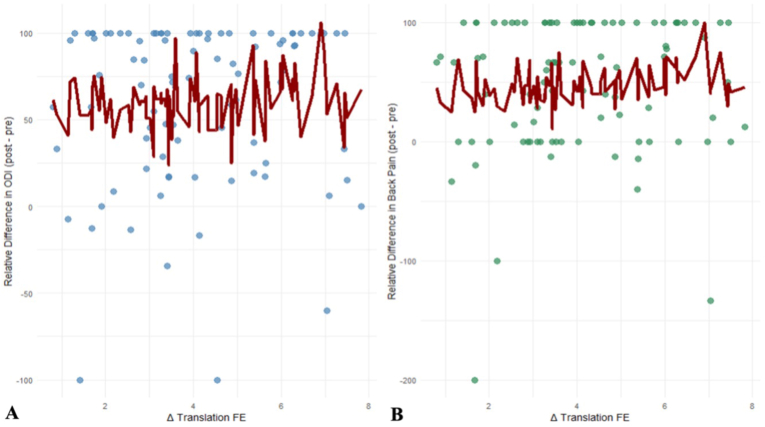
Relationship between flexion-extension radiographs (FER) translation and relative postoperative improvement in (A) functional disability (ODI) and (B) low back pain (LBP). Each dot represents an individual patient. The red line shows the predicted postoperative improvement based on the fitted regression model for the observed FER translation values.

In multivariate linear regression models adjusted for age, BMI, sex and number of levels decompressed, ΔRT_USI was identified as an independent predictor of postoperative improvement in both functional disability and LBP (p < 0.001 for both outcomes; Table 1, Fig. 3). In contrast, ΔRT_FER showed no significant association with postoperative improvement in either ODI (p = 0.698) or LBP (p = 0.341, Table 2, Fig. 4). The explanatory power of the USI-based models was substantially stronger, with an adjusted R^2^ of 0.447 for ODI and 0.214 for LBP, compared with the considerably weaker FER-based models (adjusted R^2^ = 0.053 and 0.016).

**Table 1:** Multivariable linear regression models assessing the association between upright–supine (USI)-derived relative translation and postoperative improvement in functional disability (ODI) and low back pain (LBP). Models were adjusted for age, BMI, sex and number of levels decompressed. Δ Relative Translation = difference in segmental translation between upright and supine positions. Significant values (p < 0.05) are bolded.

Predictors	β (95%-CI)	p-Value	VIF
***ΔODI_rel***			
Δ Relative Translation USI (%)	−8.53 (−10.7 to −6.36)	**< 0.001**	1.08
Age	−0.51 (−1.49 – 0.47)	0.305	1.23
BMI	−0.87 (−2.45 – 0.72)	0.279	1.25
Sex	7.77 (−7.33 – 22.88)	0.309	1.07
Number of levels decompressed	−0.41 (−8.48 – 7.66)	0.92	1.43
***ΔLBP_rel***			
Δ Relative Translation USI (%)	−7.52 (−10.66 to −4.38)	**< 0.001**	1.08
Age	−1.14 (−2.56 – 0.28)	0.144	1.23
BMI	−0.02 (−2.32 – 2.27)	0.985	1.25
Sex	−3.54 (−25.41 – 18.34)	0.749	1.07
Number of levels decompressed	1.26 (−10.43 – 12.95)	0.831	1.43

**Table 2:** Multivariable linear regression models assessing the association between flexion-extension (FER)–derived relative translation and postoperative improvement in functional disability (ODI) and low back pain (LBP). Models were adjusted for age, BMI, sex and number of levels decompressed. Δ Relative Translation = difference in segmental translation between upright and supine positions. Significant values (p < 0.05) are bolded.

Predictors	β (95%-CI)	p-Value	VIF
***ΔODI_rel***			
Δ Relative Translation FER (%)	1.33 (−4.04 – 6.7)	0.623	1.05
Age	−0.87 (−2.15 – 0.40)	0.178	1.22
BMI	−1.84 (−3.91 to −0.22)	0.079	1.23
Sex	5.22 (−14.61 – 25.05)	0.602	1.08
Number of levels decompressed	−3.81 (−14.45 – 6.83)	0.479	1.45
***ΔLBP_rel***			
Δ Relative Translation FER (%)	3.33 (−3.31 – 9.98)	0.322	1.05
Age	−1.45 (−3.03 – 0.13)	0.071	1.22
BMI	−0.77 (−3.33 – 1.78)	0.549	1.23
Sex	−6.47 (−31.01 – 18.08)	0.602	1.08
Number of levels decompressed	−2.39 (−15.57 – 10.78)	0.719	1.45

### 3.3 ROC-derived threshold of USI-derived translation and clinical improvement

**Fig. 5:**
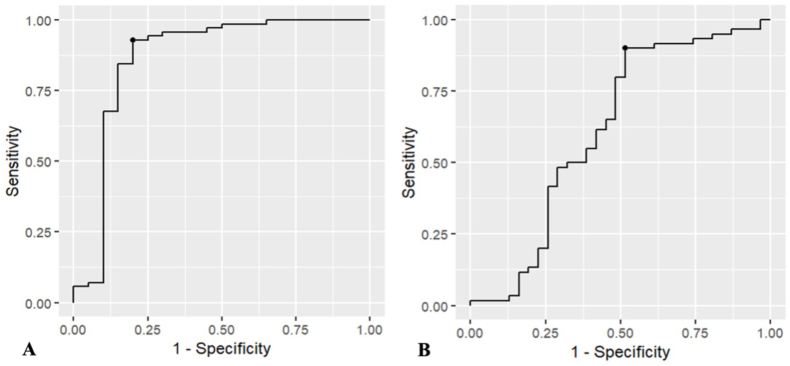
Receiver operating characteristic (ROC) curves illustrating the discriminative ability of upright–supine imaging (USI) translation for predicting achievement of the minimal clinically important difference (MCID) in (A) functional disability (ODI) and (B) low back pain (LBP). The black dot marks the optimal cutoff value, determined using the Youden index.

ROC analysis identified an optimal cutoff of 7.9% ΔRT_USI as the threshold that best discriminates patients who achieve clinically meaningful improvement. Model performance indicated excellent discrimination for ODI and moderate discrimination for LBP (Table 3, Fig. 5). Using this ROC-derived threshold, patients with ΔRT_USI ≥8% exhibited substantially less postoperative improvement in both functional disability and LBP than those below this cutoff, when undergoing decompression alone.

**Table 3:** Receiver operating characteristic (ROC) analysis of upright–supine imaging (USI)–derived relative translation for predicting achievement of the minimal clinically important difference (MCID) in functional disability (ODI) and low back pain (LBP). The optimal cutoff was determined using the Youden index. AUC = area under the curve.

Predictor	AUC	Optimal Cutoff	Sensitivity	Specificity
***MCID ODI***				
Δ Relative Translation USI (%)	0.866	7.9	0.929	0.800
***MCID LBP***				
Δ Relative Translation USI (%)	0.616	7.9	0.900	0.484

### 3.4 Assessment of segmental instability

Based on our ROC analysis, an optimal ΔRT_USI cutoff of 7.9% was identified for discriminating clinically meaningful postoperative improvement. For practical applicability, we applied a rounded threshold of ΔRT_USI ≥8%, which corresponds to the established instability cutoff used for FER (ΔRT_FER ≥8%). Using this threshold, USI identified segmental instability in 21 patients (22.8%), whereas FER revealed no cases within the analyzed cohort. Baseline demographic and surgical characteristics were comparable between the USI-stable (n = 71, 77.2%) and USI-unstable (n = 21, 22.8%) groups, except for a higher proportion of multi-level decompressions among unstable patients (p = 0.025, Table 4).

**Table 4:** Baseline demographic and surgical characteristics of the study cohort, categorized by upright–supine imaging (USI)–derived instability assessment (unstable defined as ≥8% dynamic slip). Values are presented as median (interquartile range) or n (%), as appropriate. Significant values (p < 0.05) are bolded.

Parameter		All (n = 92)	USI-Stable (n = 71)	USI-Unstable (n = 21)	p-value
***Patient demographics***					
Age [years]		69.7 (64.9, 74.2)	69.7 (64.5, 73.4)	72.0 (65.6, 76.0)	0.278
Sex	Female	40 (43.5%)	33 (46.5%)	7 (33.3%)	0.286
	Male	52 (56.5%)	38 (53.5%)	14 (66.7%)	
BMI [kg/m^2^]		28.8 (26.4, 33.5)	28.3 (26.3, 32.6)	29.8 (27.3, 35.2)	0.133
***Spondylolisthesis level***					
L3		6 (6.5%)	6 (8.5%)	0 (0.0%)	0.326
L4		85 (92.4%)	64 (90.1%)	21 (100.0%)	
L5		1 (1.1%)	1 (1.4%)	0 (0.0%)	
***Levels treated***					
Single-level		21 (22.8%)	20 (28.2%)	1 (4.8%)	**0.025**
Multi-level		71 (77.2%)	51 (71.8%)	20 (95.2%)	
Number of levels decompressed		2.0 (2.0, 3.0)	2.0 (1.0, 3.0)	3.0 (2.0, 3.0)	0.082

### 3.5 Slip dynamics across imaging modalities

The ICC demonstrated excellent agreement for both modalities, with ICC = 0.809 for ΔRT_FER and ICC = 0.941 for ΔRT_USI. Assessment of slip dynamics revealed modality-dependent differences, as ΔRT_USI significantly distinguished between stable and unstable segments (p < 0.001), whereas ΔRT_FER remained comparable across groups (p = 0.652; Table 5).

**Table 5:** Comparison of slip dynamics parameters between USI-stable and USI-unstable groups (unstable defined as ≥8% dynamic slip). Slip dynamics was defined as the difference in relative translation between imaging positions (upright–supine or flexion–extension). Data are presented as median (interquartile range). Significant values (p < 0.05) are bolded.

Parameter	All (n = 92)	USI-Stable (n = 71)	USI-Unstable (n = 21)	p-value
***Upright-Supine Imaging***				
Δ Relative Translation (%)	3.6 (1.8, 7.4)	2.6 (1.4, 4.5)	9.7 (8.7, 10.8)	**< 0.001**
***Flexion-Extension-Radiographs***				
Δ Relative Translation (%)	3.8 (2.9, 5.5)	3.6 (2.8, 5.5)	4.1 (2.9, 5.4)	0.652

### 3.6 Patient-reported outcomes according to USI-derived instability

At a median follow-up of 752 days (IQR 729–805), patients classified as USI-stable showed significantly lower postoperative ODI and LBP scores, as well as significantly greater improvement in both absolute and relative change compared with USI-unstable patients (p < 0.001 for all comparisons).

Accordingly, the proportion of patients achieving the MCID for ODI and LBP was significantly higher in the stable group than in the unstable group (p < 0.001; Table 6).

**Table 6:** Comparison of patient-reported outcome measures (PROMs) between USI-stable and USI-unstable groups. Functional disability was assessed using the Oswestry Disability Index (ODI), and back pain intensity using a numeric rating scale (NRS). The minimal clinically important difference (MCID) was defined as an improvement of ≥10 points for ODI and ≥2 points on the NRS for low back pain (LBP). Data are presented as median (interquartile range) or n (%), as appropriate. Significant values (p < 0.05) are bolded.

Parameter	All (n = 92)	USI-Stable (n = 71)	USI-Unstable (n = 21)	p-value
***Functional Disability (ODI)***				
ODI preoperative	46.0 (35.5, 56.0)	46.0 (34.0, 56.0)	46.0 (42.0, 58.0)	0.496
ODI postoperative	8.0 (0.0, 30.0)	2.0 (0.0, 16.0)	38.0 (30.0, 50.0)	**< 0.001**
ΔODI_abs	30.0 (12.0, 45.0)	36.0 (22.0, 46.0)	4.0 (−6.0, 10.0)	**< 0.001**
ΔODI_rel	76.5 (31.0, 100.0)	94.6 (69.1, 100.0)	8.7 (−12.5, 19.0)	**< 0.001**
ΔODI > MCID	71 (78.0%)	66 (94.3%)	5 (23.8%)	**< 0.001**
***Low Backpain (NRS)***				
LBP preoperative	6.0 (3.0, 8.0)	6.0 (2.0, 8.0)	7.0 (5.0, 8.0)	0.426
LBP postoperative	2.0 (0.0, 4.0)	0.0 (0.0, 2.0)	6.0 (5.0, 7.0)	**< 0.001**
ΔLBP_abs	3.0(0.0, 6.0)	4.5 (2.0, 6.8)	0.0 (−1.0, 2.0)	**< 0.001**
ΔLBP_rel	66.7 (0.0, 100.0)	74.6 (40.0, 100.0)	0.0 (−14.3, 20.0)	**< 0.001**
ΔLBP > MCID	60 (65.9%)	54 (77.1%)	6 (28.6%)	**< 0.001**
***Time to Follow Up***				
Days between surgery and FU	752.0 (729.0, 805.0)	751.0 (728.5, 803.0)	772.0 (743.0, 808.0)	0.408

## 4 Discussion

This study provides the first comprehensive comparison of USI and FER for detecting segmental instability and predicting postoperative outcomes after decompression-only surgery for DLS. The key findings are as follows: 1) ΔRT_USI was an independent predictor of postoperative improvement in both ODI and LBP, whereas ΔRT_FER showed no association with clinical outcomes; 2) ROC analysis determined a ΔRT_USI threshold of 7.9% as the optimal cutoff for distinguishing patients who achieved clinically meaningful improvement in functional disability and LBP from those who did not; 3) USI identified instability in 22.8% of patients, while the established FER threshold detected none, revealing marked modality related differences in slip dynamics assessment; 4) ΔRT_USI differed significantly between USI-stable and USI-unstable groups, while ΔRT_FER remained comparable across both; 5) Patients classified as USI-unstable demonstrated significantly less postoperative improvement in functional disability and LBP compared with USI-stable patients.

FER is used as the diagnostic standard for assessing segmental instability in DLS, providing essential guidance for surgical decision-making (Cabrera et al., 2024; Kashigar et al., 2021; Schroeder et al., 2015; Morita et al., 2020). This is highlighted by a recent cross-sectional survey by Cabrera et al. which included 479 spine surgeons and confirmed that the majority (79.1%) consider dynamic slippage on FER the most important radiographic parameter for determining operative management in DLS (Cabrera et al., 2024). The established role of FER is supported by its long-standing clinical use and incorporation into widely accepted definitions of radiographic instability. However, the practical constraints of FER, particularly its limited reproducibility, restricted applicability in patients with pain or reduced mobility, and additional radiation exposure, have prompted increasing interest in alternative approaches for dynamic assessment. Among these, USI, combining upright lateral radiographs and supine MRI, has emerged as a promising and less motion-dependent modality for evaluating segmental instability. While segmental instability is increasingly recognized as a multidimensional construct that includes parameters such as segmental angulation, facet joint effusion, disc height, and vacuum phenomena, the present study specifically focused on translational motion. This approach was chosen because translation represents the most widely used and standardized parameter for comparing dynamic imaging modalities, with established threshold values applicable to both FER and USI (Forbech Elmose et al., 2022; Folkerts et al., 2026; Sun et al., 2019).

Previous investigations demonstrated that USI detects a higher proportion of radiographic instability than FER (Liu et al., 2015; Chan et al., 2019; Viswanathan et al., 2018; Chen et al., 2018). In a prospective series of 68 patients, Liu et al. reported that USI detected instability in 42.6% of patients compared to 17.6% using FER (Liu et al., 2015). Similarly, Chen et al. observed greater translational motion and a higher incidence of instability on USI compared with FER in patients with DLS (Chen et al., 2018). The findings of the present study align with these observations, as no patient in our cohort fulfilled FER-based instability criteria, whereas USI identified instability in 21 of 92 patients (22.8%). This discrepancy likely reflects the biomechanical and practical limitations that are inherent to FER-based assessment. The diagnostic accuracy of FER depends on the patient's ability to perform adequate voluntary flexion and extension, which is frequently compromised by preoperative pain or spinal stiffness, potentially resulting in an underestimation of segmental mobility (Liu et al., 2015; Chen et al., 2018). Furthermore, reflexive or protective activation of the paraspinal muscles during weight-bearing can transiently stabilize the affected motion segment, thereby reducing observable translation and concealing underlying instability during imaging (Folkerts et al., 2026). Additionally, progressive disc collapse impairs the motion segment's ability to function as a biomechanical pivot, which further restricts detectable translation during flexion–extension maneuvers (Chen et al., 2018; Luk et al., 2003). In contrast, USI captures the positional change between axially loaded (U) and unloaded (S) conditions, allowing assessment of segmental behavior under different loading conditions without depending on active trunk motion. Although both modalities fundamentally aim to quantify the same construct, segmental instability, USI may provide a more reliable and physiologic assessment of slip dynamics by minimizing patient-dependent confounders that frequently limit the diagnostic accuracy of conventional FER.

While most previous investigations have been limited to radiographic comparisons between FER and USI, few have explored how these differences translate into postoperative outcomes (Chan et al., 2019; Viswanathan et al., 2018; Chen et al., 2018). The present study expands upon this knowledge by examining the association between imaging-derived instability and functional recovery following decompression-only surgery. In the present cohort, patients classified as USI-unstable exhibited significantly less postoperative improvement in functional disability and LBP compared with USI-stable patients. These findings confirm our initial hypothesis by demonstrating, first, that USI detects clinically relevant instability that would otherwise be missed on FER, and second, that such occult instability was associated with reduced postoperative improvement after decompression-only procedures. Although prior investigations have primarily examined patients undergoing fusion procedures, their results indirectly support our hypothesis by emphasizing the clinical significance of USI-based instability assessment (Chan et al., 2019; Viswanathan et al., 2018; Chen et al., 2018). Chen et al. demonstrated that patients with greater translational motion on USI achieved superior postoperative improvement after fusion compared with those without radiographic instability (Chen et al., 2018). Likewise, Chan et al. found that translation between U and S positions correlated more strongly with improvements in LBP and leg pain than slip dynamics parameters derived from FER (Chan et al., 2019). Similarly, Viswanathan et al. reported that patients with instability detected on USI, irrespective of FER findings, experienced greater postoperative functional recovery following fusion (Viswanathan et al., 2018). Collectively, these studies suggest two key principles: first, USI-defined instability represents a clinically meaningful marker of compromised segmental integrity that is associated with surgical outcomes; and second, while fusion effectively eliminates this instability, decompression alone leaves the mechanical component unaddressed, which may contribute to inferior outcomes. Accordingly, our results suggest that USI-stable patients may achieve favorable outcomes with decompression-only surgery, whereas USI-unstable patients appear to exhibit limited postoperative improvement. These findings support the potential role of USI as a practical tool for stratifying patients according to their expected benefit from different surgical approaches.

Building on these findings, ROC analysis identified a ΔRT_USI threshold of 7.9% as the optimal threshold for distinguishing patients with clinically meaningful postoperative improvement. This value closely corresponds to the established 8% instability criterion for FER, which has also been applied to USI in prior radiographic studies (Liu et al., 2015; Folkerts et al., 2025; Chen et al., 2018; Landi et al., 2015). However, whereas previous investigations employed this cutoff based on radiographic criteria alone, the present study provides outcome-based validation (Liu et al., 2015; Folkerts et al., 2025). The identified threshold demonstrated excellent sensitivity and specificity for ODI improvement, linking quantitative radiographic translation to functional outcomes and offering a clinically applicable reference for surgical decision-making. In the context of contemporary treatment paradigms for low-grade DLS, these findings hold substantial clinical relevance. Recent randomized trials, such as the NORDSTEN study, have shown comparable average outcomes between decompression alone and decompression with fusion, emphasizing the need for refined patient selection rather than uniform treatment strategies (Hellum et al., 2023; Austevoll et al., 2021; Luk et al., 2003). Our results suggest that USI instability, when exceeding approximately 8% ΔRT_USI, may serve as such a discriminative factor in identifying patients at higher risk for suboptimal recovery after decompression-only surgery. In this context, USI may potentially guide the indication for fusion in treatment decision making algorithms by revealing clinically relevant occult instability that FER fails to identify. Future randomized controlled trials comparing decompression-only versus decompression with fusion in patients with USI-detected occult instability are warranted to determine the clinical necessity of fusion in this specific subgroup more definitively.

Nonetheless, this study has limitations that warrant consideration. First, although the analysis was retrospective, data were derived from a prospectively collected cohort with standardized PROM assessments, which mitigates but does not eliminate the risk of bias and limits causal inference. Second, the sample size was modest, and the ΔRT_USI threshold was derived within the same cohort using ROC analysis. Although the resulting cutoff of 7.9% closely aligns with the established 8% instability threshold reported in prior literature, it remains an exploratory, internally generated estimate and therefore requires external validation before routine clinical application. Third, the analysis was limited to dynamic translational slip and did not incorporate other radiographic markers of instability, such as segmental angulation, facet joint effusion, or vacuum phenomena, which may provide complementary prognostic information. Fourth, while the models adjusted for key demographic and surgical variables, additional structural parameters were not incorporated. A parsimonious modelling strategy was intentionally applied to minimize overfitting while evaluating the independent prognostic value of dynamic translation. Residual confounding cannot be entirely excluded. Fifth, the focus on a mid-term follow-up of two years restricts the assessment of long-term outcomes, including the potential for delayed symptom recurrence or progressive instability. Sixth, while FER-defined instability was not used as an exclusion criterion, the clinical pathway leading to decompression-only surgery primarily included patients without pronounced FER-detected instability. This treatment allocation limits direct comparison of both modalities in patients with overt dynamic instability, but it reflects the clinical context in which the ability of USI to detect occult instability becomes particularly relevant. Seventh, all decompressions were performed using an open posterior laminectomy approach; extrapolation to minimally invasive or endoscopic techniques should therefore be made with caution. Finally, as a single-center study, external validity may be constrained by center-specific surgical practices and patient demographics, which could limit the applicability of these findings to broader clinical populations.

## 5 Conclusion

This study demonstrated that USI identified clinically relevant occult segmental instability that is undetected on conventional FER. Furthermore, USI-defined instability was independently associated with inferior postoperative improvement in disability and LBP following decompression-only surgery, whereas FER-derived slip dynamics parameters showed no prognostic value. The identified ΔRT_USI threshold of approximately 8% may provide an objective reference for identifying clinically meaningful instability. These findings suggest that USI may serve as a valuable modality for preoperative assessment and surgical planning in DLS.

## Consent for publication

The authors confirm that written informed consent for publication of the data included in this manuscript was obtained from all patients.

## Ethical approval

This study was approved by the Institutional Review Board of the Hospital for Special Surgery (IRB# 2015-237).

## Authors’ contribution

All listed authors made substantial contributions to the conception and design of the study, data acquisition, analysis or interpretation of the data, and/or drafting and critical revision of the manuscript, and approved the final version for submission.

TF: Conceptualization, Methodology, Data acquisition, Data curation, Formal analysis, Interpretation of data, Writing – original draft, Writing – review & editing.

JW: Conceptualization, Data acquisition, Critical revision of the manuscript, Supervision.

LS: Analysis and interpretation of data, Critical revision of the manuscript, Supervision.

AM: Conceptualization, Critical revision of the manuscript, Supervision.

BV: Conceptualization, Critical revision of the manuscript, Supervision.

PT: Conceptualization, Critical revision of the manuscript, Supervision.

PK: Analysis and interpretation of data, Critical revision of the manuscript, Supervision.

JZ: Conceptualization, Statistical analysis, Critical revision of the manuscript, Supervision.

JS: Conceptualization, Critical revision of the manuscript, Supervision.

RD: Data acquisition, Critical revision of the manuscript, Supervision.

AS: Conceptualization, Critical revision of the manuscript, Supervision.

FG: Conceptualization, Critical revision of the manuscript, Supervision.

FC: Conceptualization, Critical revision of the manuscript, Supervision.

CM: Data acquisition, Critical revision of the manuscript, Supervision.

MB: Analysis and interpretation of data, Critical revision of the manuscript, Supervision.

AH: Conceptualization, Analysis and interpretation of data, Critical revision of the manuscript, Supervision.

## Funding

The authors report no financial support or research grants were received in relation to the planning, execution, or writing of this study.

## Declaration of competing interest

The authors declare the following financial interests/personal relationships which may be considered as potential competing interests:Andrew A. Sama reports a relationship with DePuy Spine Products that includes: board membership, consulting or advisory, and speaking and lecture fees. Andrew A. Sama reports a relationship with Ortho Development Corp that includes: consulting or advisory. Andrew A. Sama reports a relationship with Clariance, Inc. That includes: board membership and consulting or advisory. Andrew A. Sama reports a relationship with Kuros Biosciences AG that includes: board membership and consulting or advisory. Andrew A. Sama reports a relationship with Vestia Ventures that includes: equity or stocks. Andrew A. Sama reports a relationship with MiRUS Investment, LLC that includes: equity or stocks. Andrew A. Sama reports a relationship with ISPH II, LLC that includes: equity or stocks. Andrew A. Sama reports a relationship with ISPH 3, LLC that includes: equity or stocks. Andrew A. Sama reports a relationship with Centinel Spine (VBros Venture Partners V) that includes: equity or stocks. Andrew A. Sama reports a relationship with Spinal Kinetics, Inc. That includes: funding grants. Frank P. Cammisa reports a relationship with Accelus that includes: consulting or advisory. Frank P. Cammisa reports a relationship with 4WEB Medical that includes: equity or stocks and funding grants. Frank P. Cammisa reports a relationship with Healthpoint Capital Partners, LP that includes: board membership and equity or stocks. Frank P. Cammisa reports a relationship with ISPH II, LLC that includes: equity or stocks. Frank P. Cammisa reports a relationship with ISPH 3 Holdings, LLC that includes: equity or stocks. Frank P. Cammisa reports a relationship with Ivy Healthcare Capital Partners, LLC that includes: equity or stocks. Frank P. Cammisa reports a relationship with Medical Device Partners II, LLC that includes: board membership and equity or stocks. Frank P. Cammisa reports a relationship with Medical Device Partners III, LLC that includes: equity or stocks. Frank P. Cammisa reports a relationship with Orthobond Corporation that includes: board membership and equity or stocks. Frank P. Cammisa reports a relationship with Spine Biopharma, LLC that includes: board membership, consulting or advisory, and equity or stocks. Frank P. Cammisa reports a relationship with Tissue Differentiation Intelligence, LLC that includes: equity or stocks. Frank P. Cammisa reports a relationship with Tissue Connect Systems, Inc. That includes: equity or stocks. Frank P. Cammisa reports a relationship with VBVP VI, LLC that includes: equity or stocks. Frank P. Cammisa reports a relationship with VBVP X, LLC that includes: equity or stocks. Frank P. Cammisa reports a relationship with Woven Orthopedic Technologies that includes: board membership and equity or stocks. Frank P. Cammisa reports a relationship with Camber Spine that includes: funding grants. Frank P. Cammisa reports a relationship with Choice Spine that includes: funding grants. Frank P. Cammisa reports a relationship with DePuy Synthes that includes: funding grants. Frank P. Cammisa reports a relationship with Centinel Spine Inc that includes: funding grants. Frank P. Cammisa reports a relationship with Royal Biologics that includes: funding grants. Federico P. Girardi reports a relationship with Lanx, Inc. That includes: consulting or advisory. Federico P. Girardi reports a relationship with Ortho Development Corp that includes: consulting or advisory. Federico P. Girardi reports a relationship with Sea Spine that includes: consulting or advisory. Federico P. Girardi reports a relationship with Centinel Spine Inc that includes: equity or stocks. Federico P. Girardi reports a relationship with BICMD that includes: equity or stocks. Federico P. Girardi reports a relationship with Healthpoint Capital Partners, LP that includes: board membership and equity or stocks. Alexander P. Hughes reports a relationship with Kuros Biosciences AG that includes: funding grants. Alexander P. Hughes reports a relationship with Expanding Innovations, Inc. That includes: funding grants. Alexander P. Hughes reports a relationship with Tissue Connect Systems, Inc. That includes: equity or stocks. Alexander P. Hughes reports a relationship with NuVasive Inc that includes: funding grants. Alexander P. Hughes reports a relationship with Globus Medical North America, Inc. That includes: funding grants. Alexander P. Hughes reports a relationship with Alphatec Spine, Inc. That includes: funding grants. If there are other authors, they declare that they have no known competing financial interests or personal relationships that could have appeared to influence the work reported in this paper.
