# Supplementary material for: Upright supine imaging can detect clinically relevant occult instability in degenerative lumbar spondylolisthesis - A two-year PROM based comparison with flexion extension radiographs
Source: Brain Spine. 2026 Apr 23;6:106064. doi: 10.1016/j.bas.2026.106064 (PMC13127177; doi:10.1016/j.bas.2026.106064)
Supplement: Supplementary file 1 [file mmc1.docx]

**Key Words:** Degenerative spondylolisthesis; Decompression surgery; Lumbar spine; Segmental instability; Flexion extension radiographs; Upright supine imaging
